# Characterization of *Acinetobacter baumannii* Isolated from Raw Milk

**DOI:** 10.3390/biology11121845

**Published:** 2022-12-18

**Authors:** Hams M. A. Mohamed, Hanan H. Abd-Elhafeez, Omar A. Al-Jabr, Mona A. El-Zamkan

**Affiliations:** 1 Department of Microbiology, Faculty of Veterinary Medicine, South Valley University, Qena 83523, Egypt; 2 Department of Cells and Tissues, Faculty of Veterinary Medicine, Assiut University, Assiut 71526, Egypt; 3 Department of Microbiology, College of Veterinary Medicine, King Faisal University, P.O. Box 400, Al-Ahsa 31982, Saudi Arabia; 4 Department of Food Hygiene and Control, Faculty of Veterinary Medicine, South Valley University, Qena 83523, Egypt

**Keywords:** *A. baumannii*, milk, CHROM agar, *rpoB*, 16S-23SrRNA, biofilm, β-lactamase genes

## Abstract

**Simple Summary:**

Although milk is a significant nutrient source for humans, it can be associated with various bacterial infections. *Acinetobacter* species can be found in milk due to residual water in milking machines, milk pipelines or coolers, the inadequate cleaning of dairy equipment, tainted udders and teats, the improper transport and storage of milk and the inadequate cleaning of dairy equipment, causing diseases. Most members of the genus *Acinetobacter* are opportunistic commensals with limited virulence and are clinically insignificant. However, *Acinetobacter* infections have recently increased in severity due to the frequent use of mechanical breathing devices, venous catheters and antibiotics, and they pose significant public health concerns. *Acinetobacter baumannii (A. baumannii)* is an opportunistic pathogen that causes various nosocomial infections. Studies using animal models and clinical data demonstrated that *A. baumannii* is a highly virulent species. It is a significant pathogen, especially due to the emergence of multidrug-resistant (MDR) strains and their association with many nosocomial infections and community-acquired infections.

**Abstract:**

*Acinetobacter baumannii (A. baumannii)* is an opportunistic pathogen associated with nosocomial infections. In this study, 100 raw milk samples were collected from Qena, Egypt, and subjected to conventional and molecular assays to determine the presence of *A. baumannii* and investigate their antimicrobial resistance and biofilm formation. Our findings revealed that, among the 100 samples, *Acinetobacter* spp. were found in 13 samples based on CHROM agar results. We further characterized them using *rpo*B and *16S-23SrRNA* sequencing and *gyr*B multiplex PCR analysis and confirmed that 9 out of the 13 *Acinetobacter* spp. isolates were *A. baumannii* and 4 were other species. The *A. baumannii* isolates were resistant to β-lactam drugs, including cefotaxime (44%), ampicillin-sulbactam and levofloxacin (33.3% for each), imipenem, meropenem and aztreonam (22.2% for each). We observed different antimicrobial resistance patterns, with a multi-antibiotic resistant (MAR) index ranging from 0.2 to 0.3. According to the PCR results, *bla_OXA-51_* and *bla_OXA-23_* genes were amplified in 100% and 55.5% of the *A. baumannii* isolates, respectively, while the *bla_OXA-58_* gene was not amplified. Furthermore, the metallo-β-lactamases (MBL) genes *bla_IMP_* and *bla_NDM_* were found in 11.1% and 22.2% of isolates, respectively, while *bla_VIM_* was not amplified. Additionally, eight *A. baumannii* isolates (88.8%) produced black-colored colonies on Congo red agar, demonstrating their biofilm production capacity. These results showed that, besides other foodborne pathogens, raw milk should also be examined for *A. baumannii*, which could be a public health concern.

## 1. Introduction

Milk is a significant nutrient source for humans, but drinking unsafe milk might cause various bacterial infections [1,2], including those by *Acinetobacter*. These foodborne pathogens might potentially cause human diseases (Malta et al., 2020). The most common sources of *Acinetobacter* in milk include residual water in milking machines, milk pipelines or coolers, the inadequate cleaning of dairy equipment, tainted udders and teats, the improper transport and storage of milk and the inadequate cleaning of dairy equipment [3].

*Acinetobacter* species are gram-negative, non-fermenting, aerobic, non-motile, catalase-positive, indole-negative and oxidase-negative bacteria [4,5]. The ideal temperature range for most strains for converting nitrates into nitrites is 33–35 °C [6,7]. They can resist dryness [8] and disinfectants such as phenols and chlorhexidine [9].

Some Acinetobacter spp. can survive temperatures up to 75 °C [7], and this is taken into consideration because, although ultra-heat treatments are effective in eliminating many microbes, there is still much public debate regarding the potential benefits of the high popularity of raw milk consumption. [10]. In addition, some countries, including Egypt and the United States, still prefer to consume locally manufactured dairy products made from raw milk, such as kareish and traditional artisan raw cheddar cheese [11,12].

Although most *Acinetobacter* members are opportunistic commensals with limited virulence and negligible clinical importance, infections caused by *Acinetobacter* spp. have increased in severity with the continuous use of mechanical breathing devices and venous catheters and, particularly, the increased use of antibiotics [13].

*Acinetobacter* can adapt to challenging environmental conditions and potentially develop resistance against several antibiotic classes, making it a significant public health concern. In particular, *A. baumannii* is associated with most *Acinetobacter* infections, followed by *A. calcoaceticus* and *A.lwoffii*. Other species include *A. haemolyticus*, *A. johnsonii*, *A. junii*, *A. nosocomialis*, *A. pittii*, *A. schindleri* and *A. ursingi*. Studies using animal models and clinical data demonstrated that *A. baumannii* is the most virulent species [14]. Although it is ubiquitous, the frequency of the occurrence of the pathogenic species from this genus in food sources and drinking water is not known yet [15].

*A. baumannii* is a significant pathogen due to the emergence of multidrug-resistant (MDR) strains and their association with nosocomial infections and community-acquired infections. The Infectious Diseases Society of America has considered *A. baumannii* an ESKAPE pathogen, a category which includes *Enterococcus faecium*, *Staphylococcus aureus*, *Klebsiella pneumoniae*, *A. baumannii*, *Pseudomonas aeruginosa* and *Enterobacter* spp. Escape pathogens are mainly responsible for nosocomial infections worldwide and can resist different antibiotics [16].

Recently, due to the fast acquisition of several antibiotic-resistance genes, particularly for β-lactam antibiotics, MDR *A. baumannii* has been associated with high morbidity and mortality in children and has challenged conventional therapeutics (e.g., penicillins, cephalosporins, carbapenems and monobactams) [17].

Carbapenems are effective against bacterial pathogens that are resistant to extended-spectrum β-lactamases, such as MDR *A. baumannii* (ESBL) [18]. However, with the increased use of carbapenems, new carbapenem-hydrolyzing β-lactamases have emerged, increasing the chance of treatment failure. Class B metallo-β-lactamases (MBLs), such as IMP (imipenemase), VIM (Verona integrin-encoded MβL) and NDM (New Delhi MβL), class C AmpC cephalosporinases, class D carbapenemases/oxacillinases and OXA types have been observed in *A. baumannii*, including OXA-23-like, OXA-24-like, OXA-51-like and OXA-58-like. [19,20].

*Acinetobacter* spp. are biofilm producers that can acquire and transfer resistance genes, enhancing their antibiotic resistance ability [21]. Studies have shown that clinical isolates can produce biofilms more efficiently than environmental isolates and that there is a substantial correlation between biofilm production and multiple-drug resistance [22,23,24].

Therefore, in this study, we identified *A. baumannii* in milk samples and investigated their ability to form biofilms and antimicrobial susceptibility to different families of antibiotics. We also detected OXA and MBL genes responsible for carbapenem resistance.

## 2. Materials and Methods

### 2.1. Milk Sampling

We collected 100 raw milk samples from different markets in the Qena provinces between December 2020 and April 2021. The samples were collected in a sterile snap cap milk collection vial, placed in ice-cooled containers and processed within 24 h of collection.

### 2.2. Isolation of Acinetobacter spp.

Each milk sample (10 µL) was streaked on the chromogenic culture media *Acinetobacter* (CHROM^TM^ agar *Acinetobacter* Media Pioneer, Paris, France) and incubated at 37 °C for 48 h. The suspected red *Acinetobacter* colonies were further subcultured on MacConkey agar (Oxoid, Basingstoke, UK) and then on Tryptic soy agar (Lab, Neogen Company, Rochdale, UK) at 37 °C for 24–48 h for purification [2]. The suspected isolates were used for staining and other biochemical assays, including catalase, oxidase, citrate, nitrate reduction, arginine hydrolysis, glucose fermentation, hemolysis, the reaction on the triple sugar iron and the motility test, according to Constantiniu et al. [25].

### 2.3. Genotypic Identification of A. baumannii

*Acinetobacter* spp. were identified based on Gurung et al. [2]. Briefly, the *rpo*B gene of *Acinetobacter* was amplified and sequenced using two primer sets, as previously described by La Scola et al. [26]. The *16S-23S rRNA* gene intergenic spacer regions of the positive *rpoB* isolates were amplified and sequenced (Supplementary Table S1) to identify the *Acinetobacter calcoaceticus–A. baumannii* complex, as described previously [27]. Finally, *A. baumannii* was differentiated from the *A*. *Calcoaceticus–A. baumannii* complex by the DNA gyrase subunitB (*gyr*B)-based Multiplex Polymerase Chain Reaction method, as described by Higgins et al. [28]. The sequences of primers are described in Supplementary Table S1.

### 2.4. Antimicrobial Susceptibility Testing

*Acinetobacter’s* antimicrobial susceptibility test was conducted using the agar disc diffusion method based on the Clinical and Laboratory Standards Institute (CLSI) guidelines [29]. Mueller–Hinton agar was used (Oxoid, CM0337, Basingstoke, UK). The antibiotics examined included amikacin (30 g/disc), gentamicin (10 g/disc), ampicillin-sulbactam (10 g/disc), piperacillin (100 g/disc), cefotaxime (30 g/disc), cefepime (30 g/disc), imipenem (10 g/disc), meropenem (10 g/disc) and ciprofloxacin (5 μg/disc). The zone diameter breakpoints (mm) were interpreted according to CLSI [26] and the European Committee on Antimicrobial Susceptibility Testing (EUCAST) [30]. The Multi-Antibiotic Resistant (MAR) index is calculated based on the number of antibiotics that an isolate is resistant to (a) divided by the total number of antibiotics utilized in the study (b) [31], using the equation shown below:

MAR Index = a/b

### 2.5. Screening of Metallo-β-lactamases Production Using the Disk Method

Indicator strain: E. coli (ATCC 29522) was obtained from the Faculty of Agriculture at Ain Shams University in Giza, Egypt.

A suspension of a tested *A. baumannii* isolate was made by suspending a full 10 μL inoculation loop of a freshly cultured *A. baumannii* isolate, taken from a blood agar plate in 400 μL of water. Consequently, the test disc, which contained 10 g of meropenem, was dipped in the suspension and incubated at 35 °C for two hours. The disc was taken out of the suspension using an inoculation loop and put on a Mueller–Hinton agar plate, which was cultured with susceptible *E. coli* (ATCC 29522) that was prepared as a suspension of 0.5 a McFarland at OD595 and was then incubated at 35 °C. The susceptibility disk’s meropenem was rendered inactive, allowing the *E. coli* to grow unhindered; this means that the isolates were able to produce carbapenemases. A clear inhibitory zone was produced after 24 h with discs that were incubated in phosphate puffer saline (PBS) as a negative control devoid of carbapenemases [32].

### 2.6. Detection of Metallo-β-lactamasesgenes

Multiplex PCR was performed to investigate the presence of carbapenemases, including OXA-type, *bla_OXA-51-like_, bla_OXA-23-like_* and *bla_OXA-58-like_*, based on a previous study [33]. Amplification was carried out using a final volume of 25 µL, consisting of 1X PCR buffer (Qiagen, Hilden, Germany), 1U Taq polymerase (Roche, Meylan, France), 2 mMMgCl_2_, 200 mM of Deoxynucleo-tide triphosphate (dNTP) (Biotools, Madrid, Spain), 0.2 mM of each primer (Pharmacia Biotech, Piscataway, NJ, USA) and 1 µL of template DNA. The PCR was carried out using a thermal cycler (Hamburg, Germany) under the following conditions: 94 °C for 5 min, followed by 30 cycles at 94 °C for 30 s, 53 °C for 40 s and 72 °C for 50s, then final extension at 72 °C for 6 min. The PCR products were separated by electrophoresis on 1.5% agarose gels (Synacron) and visualized under a UV gel documentation system after staining with ethidium bromide.

According to Kazi et al. [34] the genes *bla_IMP_*, *bla_VIM_* and *bla_NDM_* were amplified using uniplex PCR. The amplification was conducted using a final volume of 25 μL containing: 12.5 μL of PCR Mastermix (Emerald Amp GT), 1 μL for each primer (Table 1), 1.5 μL of the DNA template and 9 μL of PCR-grade water using a thermal cycler (MJ Research, Inc. Watertown). The optimal cycling conditions were 94 °C for 3 min, followed by 29 cycles of 94 °C for 30 s, 60 °C for 30 s and 72 °C for 30 s, with a final extension of 72 °C for 5 min. The bands were detected on 2% agarose gel electrophoresis.

### 2.7. PCR Positive Control

*Pseudomonas aeruginosa* isolates (ATCC 27853) were used as a positive control and were obtained from the Animal Health Research Institute, Dokkki, Egypt.

### 2.8. Qualitative and Quantatine Methods for the Detction of Biofilm Formation

#### 2.8.1. Congo Red Agar

According to Freeman et al. [35], we prepared Congo red agar using 15 g/L nutrient agar (Oxoid, Thermo Scientific^TM^, Waltham, MA, USA, CM0003B), 37 g/L sucrose (Oxoid) and 0.8 g/L Congo Red (Merck, Darmstadt, Germany). It was stored for up to 48 h and used to measure biofilm production. Biofilm-producing colonies appear as black colonies and can also stain the medium due to exopolysaccharide synthesis [36,37].

#### 2.8.2. Microtiter Plate Technique

This technique was performed according Stepanović et al. [38] Briefly, a brain–heart infusion broth (BHI) (Oxoid, Waltham, CA, USA) was inoculated with A. baumannii strains and was left to grow for 24 h at 37 °C. OD was adjusted to an OD600 of 1 ± 0.05, and then the bacteria were diluted 1:100 with sterile BHI with 1% glucose (Oxoid) before being put in a 96-well polystyrene microplate containing 200 µL of BHI medium and then being incubated overnight at 37 °C. The negative control wells only used the medium. The P. aeruginosa ATCC 27853 reference strain was the positive control for biofilm formation. After incubation, the media and the majority of the bacteria were quickly removed from the plates after incubation. A total of 200 µL of PBS 1× was introduced into the wells using a pipette, and after that, the plate was tilted to remove the liquid for pipette-based washing. Washing was repeated two more times. Using crystal violet staining (0.5%), which involves pouring 150 µL of dye into each well and letting them sit at room temperature for 5 min, biofilm formation was assessed. The extra dye was washed out using a pipette. After the plates had air-dried, the leftover dye was dissolved with 200 µL of glacial acetic acid (33% *v*/*v*) in each well. Using the ELISA auto-reader (Thermo Fisher Multiskan^TM^ FC), staining was measured at OD620. The criteria for categorized biofilm formation were as follows: biofilm was not formed if OD ≤ ODc (negative), it was weak if ODc < OD < 2 × ODc and it was moderate if 2 × ODc < OD < 4 × ODc. A biofilm was considered strong at 4 × ODc < OD.

## 3. Results

### 3.1. Results of the Isolation and Identification of A. baumannii

In this study, out of 100 milk samples collected, 13 were suspected to be contaminated with *Acinetobacter* spp. based on the CHROM agar observations. Microscopic and biochemical examination showed the presence of gram-negative cocci that were catalase-positive and negative for oxidase and nitrate reduction tests. Moreover, negative results for hemolysis and motility. The isolates showed alkaline reaction in triple sugar iron (K/K) and variable results with citrate, arginine hydrolysis and the glucose fermentation test were demonstrated (Supplementary Table S2).

Furthermore, we used molecular identification to identify different *Acinetobacter* species by amplifying and sequencing genes, including *rpo*B, *16S-23Sr*RNA, and *gyr*B. The results showed that certain *rpo*B genes were amplified in the suspected 13 isolates. Finally, these isolates were classified as *A. baumannii* (nine isolates), *A.pitti* (two isolates), *A. rudis* (one isolate) and *A. oryzae* (one isolate) (Table 1). We observed 99% similarity in the *rpo*B gene sequences from all strains using pairwise comparison. Further, the intergenic spacer region of the *16S-23Sr*DNA was sequenced to identify the *A. calcoaceticus–A. baumannii* complex, and the results showed that, of the 13 *Acinetobacter* isolates, 11 belonged to the *A. calcoaceticus–A. baumannii* complex, 9 were *A. baumannii* and 2 were A. pitti (Table 1).

**Figure 1:**
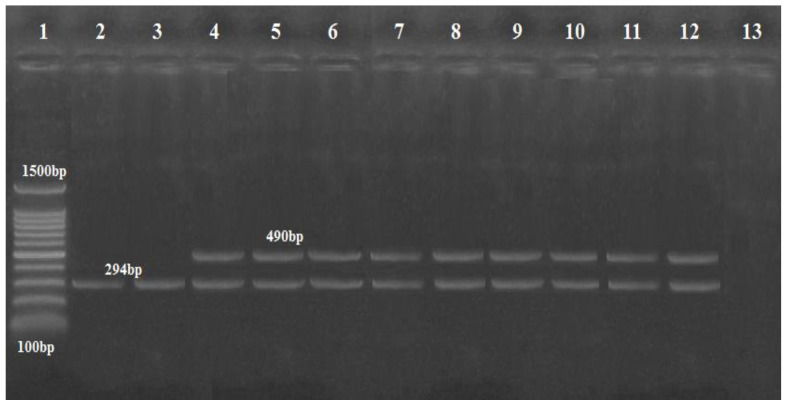
Agarose gel image showing *Acinetobacter* isolates differentiated by multiplex PCR using *gyr B*-primers. Lane: DNA Marker (100 bp ladder Gold Bio Company, Cat.D001, US) lanes (4–12) were *A. baumannii* at 490 bp; lanes (2 and 3) were *Acinetobacter* genomic sp. 13TU (394 bp); lane 13: negative control with no DNA template.

Multiplex PCR was performed using three primers for the *gyr*B gene for both *A. baumannii* and genomic sp. 13TU, and in 11 isolates, a 294-base-pair (bp) amplicon (sp4F to sp4R) was observed, whereas 9 *A. baumannii* isolates produced the second amplicon of 490 bp (sp2F to sp4R) (Figure 1).

### 3.2. Antimicrobial Susceptibility Profile and MBL Production of A. baumannii Isolates

The antimicrobial susceptibility of *A. baumannii* (n = 9) was tested against 12 antibiotics (Table 2). However, *A. baumannii* isolates showed sensitivity to some antibiotics such as amikacin (100%) and ciprofloxacin (88.8%); some isolates exhibited resistance against cefotaxim (44.4% for each), Ampicillin-sulbactam, levofloxacin (33.3% for each), imipenem, meropenem, piperacillin and aztreonam (22.2% for each). 

Some *A. baumannii* isolates showed five distinct antimicrobial resistance patterns: 1. levofloxacin, ampicillin-sulbactam, imipenem and aztreonam with MAR (0.33); 2. ciprofloxacin, piperacillin, cefotaxime and meropenem with MAR (0.33); 3. ampicillin-sulbactam, cefotaxime and cefepime with MAR (0.22); 4. levofloxacin, tetracycline, gentamycin and cefotaxime with MAR (0.33); 5. piperacillin, cefotaxime, cefepime and meropenem with MAR (0.33) (Table 3).

**Figure 2:**
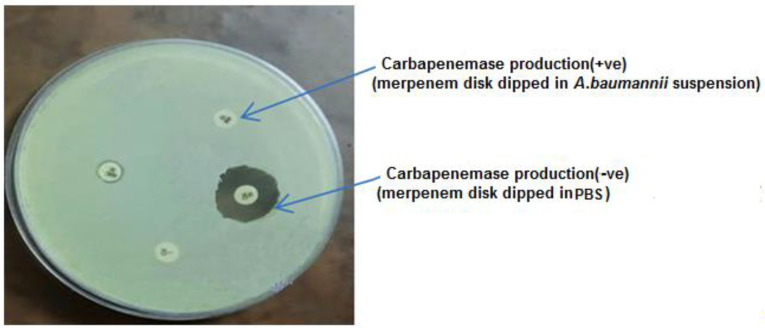
Ability of *A. baumannii* for Carbapenemase production.

Even though the data clearly indicated some resistance of *A*. *baumannii* isolates to β-lactam antibiotics, including penicillin, cephalosporin and carbapenem, we focused on carbapenem resistance because 44.4% (4\9) of isolates showed resistance for imipenem and meropenem. The phenotypic results of the MBL test confirmed the production of carbapenemase in these four out of nine isolates (Figure 2).

### 3.3. PCR Results of the Carbapenem Resistance Genes

**Figure 3:**
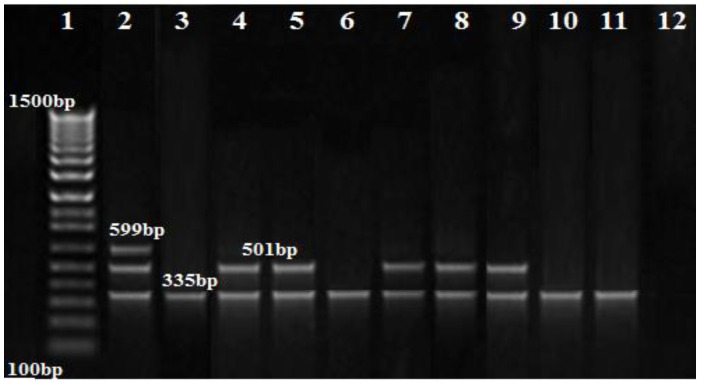
Agarose gel image showing the separation of the OXA gene products amplified using multiplex PCR. Lane 1: DNA marker (100 bp ladder Biolabs Company, Cat.N3231S, London, England), lane 2: positive control, lane 3–11: positive for the *bla_OXA51_* gene (335 bp), lane 4,5,7,8,9: positive for the *bla_OXA23_* gene (501 bp); no band was seen for *bla_OXA58_* (599 bp).

**Figure 4:**
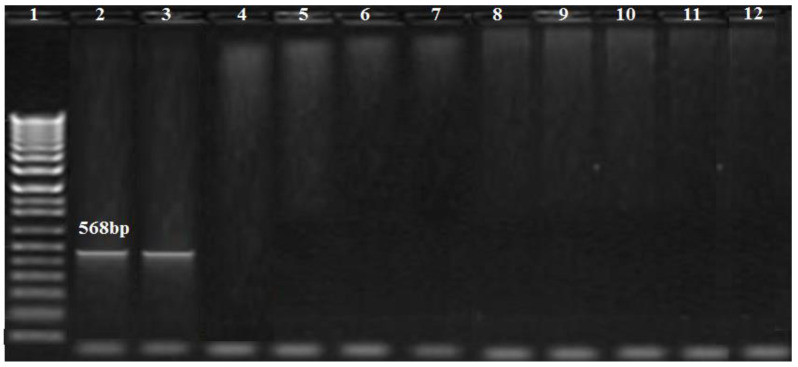
Amplification profile of the *bla_IMP_* gene (568 bp). Lane 1: DNA marker (100 bp Biolabs Company, Cat.N3231S, London, England); lane 2: positive control; lane 3: positive for the *bla_IMP_* gene; lane 12: negative control.

**Figure 5:**
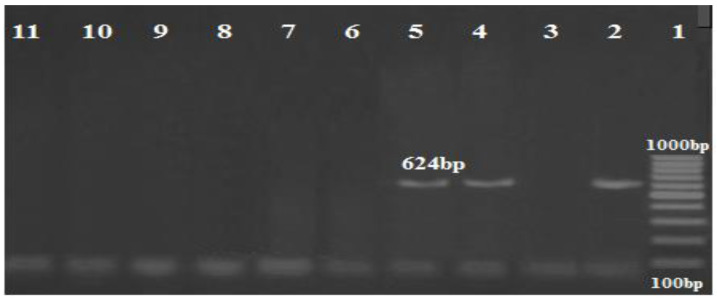
Amplification profile of the *bla_NDM_* gene (624 bp). Lane 1: DNA marker (100 bp ladder GoldBio Company, Cat.D001, Olivette, Mo, USA); lane 2: positive control; lane (4,5): positive for the *bla_NDM_* gene; lane 11: negative control.

The PCR results showed that *bla_OXA-51_* and *bla_OXA-23_* genes were amplified in nine (100%) and five (55.5%) of the *A. baumannii* isolates, respectively, while the *bla_OXA-58_* gene was not amplified in any isolate (Figure 3). Additionally, the MBL genes, *bla_IMP_*_,_ and *bla_NDM_*, were found in one (11.1%), and two (22.2%) isolates, respectively (Figure 3, Figure 4 and Figure 5), while *bla_VIM_* was not amplified in any of them.

### 3.4. Biofilm Formation of A. baumannii Isolates

**Figure 6:**
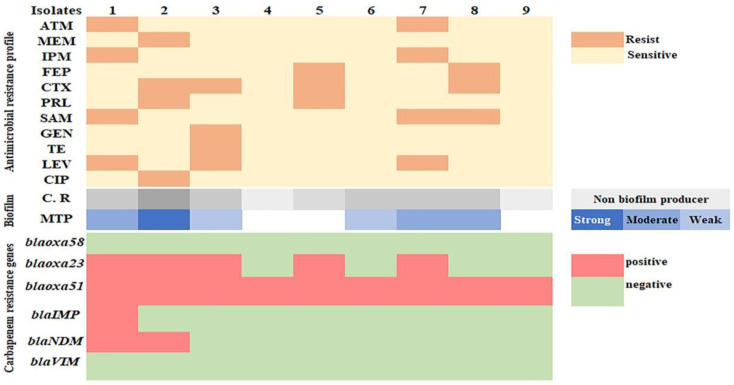
Heat map showing the antimicrobial resistance and biofilm formation ability of *A. baumannii* isolates.

Furthermore, eight *A. baumannii* isolates (88.8%) produced black colonies on Congo red agar, demonstrating their biofilm-producing capacity, while just six isolates (66.6%) were able to form biofilm (one strong, three moderate and two weak biofilm formations) on the microtiter plate method (Figure 6 and Supplementary Table S3).

## 4. Discussion

Recently, *A. baumannii* has been significantly linked to several infections, including catheter-related infections, meningitis, bacteremia, soft-tissue infections, peritonitis and endocarditis, in intensive care units (ICU) due to its extraordinary capacity to obtain or enhance the antimicrobial resistance factors. Therefore, scientists are currently interested in discovering this novel, antibiotic-resistant strain [39].

We identified *Acinetobacter* spp. in 13 milk samples on CHROM agar media. Several authors have suggested using CHROM agar as a quick and easy medium for detecting *Acinetobacter*, as it contains chromogenic substrates cleaved by *Acinetobacter* spp. enzymes, resulting in unique, red-colored *Acinetobacter* colonies. This increases the efficiency of infection control procedures, decreases the time required to administer the right antibiotic therapy to the infected patients and, hence, lowers mortality [2,40].

However, further information about the interspecies relationships among the genus *Acinetobacter* is needed. It is challenging to distinguish between the *Acinetobacter* species, as they share several phonotypical traits. There are several methods for identifying the pathogenic *Acinetobacter* strains. Even though PCR is the preferred method for identifying *Acinetobacter* species, the sequencing of several genes is required for species-level identification [26]

In this study, due to the close relationship between *Acinetobacter* genomic species, especially those belonging to the *A. calcoaceticus–baumannii* complex, we used different primers to amplify and sequence target genes, such as *rpoB, 16S-23S* and *gyrB*, to identify *Acinetobacter* spp., especially *A. bummanii*. Several studies have found that the sequencing of the *rpoB* gene and the *16S-23S rRNA* gene spacer region enables the identification of *Acinetobacter* isolates at the species level [2,26,41,42,43], *A. bummanii* and the 13TU genomic species showed interspecies variability, which could be discriminated by amplifying the *gyr*B gene [2,28,44].

We performed molecular identification to confirm the presence of *Acinetobacter* spp. DNA in 13 isolates. Among them, eight were identified as *A. bummanii*. A previous study [45] illustrated that 18 isolates of *Acinetobacter* species were isolated from 120 raw milk samples; among them, 12 were *A. bummanii*. Additionally, Gurung et al. [2] found that out of 2287 bulk milk samples, *Acinetobacter* spp. were isolated from 176 bulk samples. Among them, 57 were *A. bummanii.* Moreover, Jayarao et al. [46] identified *Acinetobacter* spp. in 28 out of 205 isolates from bulk milk samples. Additionally, Ndegwa et al. [47] found that 10 isolates of *Acinetobacter* species were identified from 21 goat milk samples in Kenya. Due to these varied results, we used various selective methods to isolate *Acinetobacter* spp. and to clarify the prevalence of *Acinetobacter* spp. as compared with these previous studies.

Infections by antibiotic-resistant bacteria, especially multi-resistant bacteria, are challenging to treat, resulting in serious health issues and even death due to extended hospital stays and unsuccessful treatment attempts [48].

In our study, some *A. baumannii* isolates showed resistance to different antibiotics. This is consistent with previous studies [49,50] which found that clinical *A. bumannii* isolates from humans were highly resistant to fluoroquinolone, amino-glycosides, cephalosporin and carbapenem. The resistance of some *A. bumannii* isolates is attributed to the AdeR–AdeS complex, encoded by the adeRS operon, which contributes to acquired antibiotic resistance against different antibiotics [51].

Another factor that might contribute to the resistance found in this study is the selection pressure exerted by using these antimicrobial drugs to treat sick cattle [49]. However, Gurung et al. [2] observed that most *Acinetobacter* isolates obtained from bulk milk tank samples were susceptible to several antibiotics. They hypothesized that this was because milk is generally extracted from healthy cattle, not those treated with antimicrobial agents.

Antibiotic resistance patterns varied among the nine *A. bummannii* isolates with an MAR index higher than 0.2. This is considered an indicator of serious contamination in milk, which is linked to the excessive use of antibiotics in animal treatments, which resulted in the creation of strains resistant to a number of antibiotics [52].

The carbapenem resistance observed in these isolates is a significant issue because carbapenem is generally used as an alternative for other β-lactam drugs, including penicillin and cephalosporins [53]. This resistance was mediated by several carbapenemase genes. OXA-type genes, which mainly cause carbapenem resistance in *A. baumannii*, are intrinsic and can be located on chromosomes and plasmids. The gene *blaoxa* encoded and expressed OXA-like enzymes and significantly increased carbapenem resistance [54,55].

The PCR results showed that *bla_OXA-51-like_* was amplified in all *A. baumannii* isolates, while *bla_OXA-23-like_* was amplified only in 55.5% of isolates. These findings were almost identical to previous studies [39,56], showing that 100% of *A. baumannii* had *bla_OXA-51-like_* genes, while 90% were positive for *bla_OXA-23-like_* genes. [57] The ubiquitous nature of OXA-51 in *A. baumannii* has made it a critical genetic marker for identifying *Acinetobacter* at the species level. Turton et al. [58] found that the *bla_OXA51-like_* genes played an important role in the identification and differentiation of *A. bummannii* from other *Acinetobacter* spp., and this explains the high incidence of the *bla_OXA-51-like_* genes compared to other genes in our study. Another study [59] discovered that the ability of OXA-51-like proteins to hydrolyze β-Lactam antibiotics, including penicillins (benzylpenicillin, ampicillin, ticarcillin and piperacillin) and carbapenems (imipenem and meropenem), was supported by the expression of other genes. However, *bla_OXA-23-like_* proteins were primarily responsible for imipenem resistance, and its plasmid location enhanced the possibility of the horizontal transfer of resistance.

Our findings also revealed that the *bla_IMP_* and *bla_NDM_* genes were amplified in one and two isolates, respectively, while the *bla_VIM_* gene was not amplified. However, previous studies reported that the *bla_IMP_* and *bla_VIM_* genes were not amplified in the isolates [34]. A study showed that the Verona integron-encoded MBL (VIM) genes were found to be extremely infrequent in Enterobacteriaceae [60], while another [61] discovered that MBL oxacillinases and *bla_NDM1_* are the primary causes of carbapenem resistance.

Based on the MBL phenotypic screening results, 44.4% of *A. baumannii* isolates produced MBL. Despite phenotypic testing, only one amplicon for the *bla_imp_* gene was found among the studied MBL genes. This might be due to the presence of unidentified hereditary determinants, which PCR might not detect, as the PCR primers were only created for specific known genetic positions. Contrastingly, these results might be true-positive for other MBL genes [62].

*A. baumannii* can efficiently produce biofilms that enable it to tenaciously proliferate under challenging circumstances and habitats. *A. baumannii* can produce biofilms on biotic surfaces, including epithelial cells, and on abiotic surfaces, including glass and the medical equipment used in ICUs. Most of the *A. baumannii* isolates from our study were consistent with the findings Malta et al. [63], who found that 100% of *A. baumannii* isolated from goat milk formed biofilm. Several studies have also emphasized the severity of *A. baumannii* biofilm-associated infections, such as ventilator-associated pneumonia and catheter-related infections, both of which are resistant to antibiotic therapy and are most frequently caused by *A. baumannii* biofilms. [21,64,65].

The biofilm matrix around the bacterial cells enables them to withstand harsh conditions and promotes the propagation of antibiotic-resistance genes. Therefore, the current drugs that are available for treating infections caused by *A. baumannii* biofilms are ineffective [66]. Here, a microtiter plate test overcame the problem of false-positive results in two isolates on Congo red agar; these results are reinforced by Melo et al. [67], who recorded that that sensitivity of the Congo red agar test was 88.9%, while that of the microtiter plate test was 100%. Gaddy and Actis [68] found that *A. baumannii* can efficiently produce biofilms due to the presence of pili, outer membrane proteins and macromolecular secretions. When pili adhere to abiotic surfaces, they initiate the formation of microcolonies, followed by the formation of fully developed biofilms.

## 5. Conclusions

In conclusion, the detection of *A. baumannii* and its antimicrobial resistance is critical for controlling infections. Efficient molecular techniques, such as DNA sequencing, enable faster identification than other traditional techniques and can readily differentiate between closely related species, facilitating immediate prevention. Therefore, these techniques are necessary for the early detection of these microbes. *A. baumannii* isolates showed resistance to several antibiotics with MAR indices higher than 0.2, indicating that these are potentially hazardous to consumers. The antibiotic resistance in *Acinetobacter* spp. still needs further studies, especially the *bla_OXA51_* gene, because the role of this gene in antibiotic resistance is linked to the mechanism of other genes. In addition to that, it has a major role in differentiating *A. baumannii* from other species.

## Figures and Tables

**Table 1:** Accession numbers of *Acinetobacter* spp. for sequenced *rpoB* and *16S-23S* ribosomal RNA intergenic genes.

Strains	*rpoB Gene*			*16S-23SrRNA Intergenic Space*	
Strain Name on Gene Bank	Spacer Region Zone 1	Spacer Region Zone 2	Identified Strains	Accession Number	*Identified A. baumannii–A. calcoaceticus Complex*
AcbaQHM_1	OP326286	OP326299	*A. baumannii*	OP321175	*A. baumannii*
AcbaQHM_2	OP326287	OP326300	*A. baumannii*	OP321173	*A. baumannii*
AcbaQHM_3	OP326288	OP326301	*A. baumannii*	OP321177	*A. baumannii*
AcbaQHM_4	OP326289	OP326302	*A. baumannii*	OP321174	*A. baumannii*
AcbaQHM_5	OP326290	OP326303	*A. baumannii*	OP321176	*A. baumannii*
AcbaQHM_6	OP326291	OP326304	*A. baumannii*	OP321178	*A. baumannii*
AcbaQHM_7	OP326292	OP326305	*A. baumannii*	OP321258	*A. baumannii*
AcbaQHM_8	OP326293	OP326306	*A. baumannii*	OP321257	*A. baumannii*
AcbaQHM_9	OP326294	OP326307	*A. baumannii*	OP321259	*A. baumannii*
AcbiQHM_10	OP326295	OP326308	*A. pittii*	OP321256	*A. pittii*
AcbiQHM_11	OP326296	OP326309	*A. pittii*	OP321255	*A. pittii*
ACspQHM_12	OP326297	OP326310	*A.oryzae*	-------	-----
AcspQHM_13	OP326298	OP326311	*A.rudis*	------	------

**Table 2:** Antimicrobial susceptibility profiles of *A. baumannii* isolates.

Antibiotic Classes	Antimicrobial	Susceptible Isolates	Resistant Isolates
Fluoroquinolones	Ciprofloxacin Levofloxacin	8 (88.8%) 6 (66.6%)	1 (11.1%) 3 (33.3%)
Tetracyclines	Tetracycline	8 (88.8%)	1 (11.1%)
Aminoglycosides	Amikacin Gentamicin	9 (100%) 8 (88.8%)	0 (0%) 1 (11.1%)
Penicillin	Ampicillin-sulbactam Piperacillin	6 (66.6%) 7 (77.7%)	3 (33.3%) 2 (22.2%)
Monobactams	Aztreonam	7 (77.7%)	2 (22.2%)
Cephalosporins	Cefotaxime Cefepime	5 (55.5%) 7 (77.7%)	4 (44.4%) 2 (22.2%)
Carbapenems	Imipenem Meropenem	7 (77.7%) 7 (77.7%)	2 (22.2%) 2 (22.2%)

**Table 3:** Antimicrobial resistance patterns of *A. baumannii* isolates.

Antibiotic Patterns	No. of Isolates	MAR Index
levofloxacin, ampicillin-sulbactam, imipenem, aztreonam	2	0.33
ciprofloxacin, piperacillin, cefotaxime, meropenem	1	0.33
ampicillin-sulbactam, cefotaxime, cefepime	1	0.20
levofloxacin, tetracyclins, gentamycin, cefotaxime	1	0.33
piperacillin, cefotaxime, cefepime, meropenem	1	0.33

## Data Availability

The results and analyses presented in this paper are freely available upon request from Hams M.A. Mohamed and Mona A. El-Zamkan.

## Supplementary Materials

The following supporting information can be downloaded at: https://www.mdpi.com/article/10.3390/biology11121845/s1, Supplementary Tabel S1: Primers sequence, Supplementary Table S2: Biochemical profile of *Acintobacter* spp., Supplementary Table S3: Results of biofilm formation by microtiter plate (OD600)).
