# Supplementary material for: Characterization of Acinetobacter baumannii Isolated from Raw Milk
Source: Biology (Basel). 2022 Dec 18;11(12):1845. doi: 10.3390/biology11121845 (PMC9775129; doi:10.3390/biology11121845)
Supplement: Supplementary file 1 [file biology-11-01845-s001.zip › biology-2068553-supplementary.pdf]

Supplemantry Tabel S1:Primers sequence

gene	Primer sequence(5′ → 3′)	Amplicon	References
***rpoB* gene**	Ac696F TAYCGYAAAGAYTTGAAAGAAG<br>Ac1093R CMACACCYTTGTTMCCRTGA	397	[26]
	Ac1055F GTGATAARATGGCBGGTCGT<br>Ac1598R CGBGCRTGCATYTTGTCRT	544	
16S-23S rRNA	A15 FAGTCGTAACAAGGTAGCCG<br>B15 RC T/C A/G T/C TGCCAAGGCAT CCACC	695	[27]
gyr B	sp4FGTTCCTGATCCGAAATTCTCG<br>sp4RAACGGAGCTTGTCAGGGTTA	490	[28]
	sp4F CACGCCGTAAGAGTGCATTA<br>sp4R AACGGAGCTTGTCAGGGTTA	294	
***bla*OXA-51- like**	TAATGCTTTGATCGGCCTTG<br>TGGATTGCACTTCATCTTGG	353	
***bla*OXA-23- like -**	GATCGGATTGGAGAACCAGA<br>ATTTCTGACCGCATTTCCAT	501	[66]
***bla*OXA-58- like**	AAGTATTGGGGCTTGTGCTG<br>CCCCTCTGCGCTCTACATAC	599	
***bla*IMP**	TCGTTTGAAGAAGTTAACGG<br>ATGTAAGTTTCAAGAGTGATGC	568	[68]
***bla*VIM**	GGTGTTTGGTCGCATATCGCAA<br>ATTCAGCCAGATCGGCATCGGC	502	
***bla*NDM**	GGTTTGGCGATCTGGTTTTC 624<br>CGGAATGGCTCATCACGATC	624	[62]

Supplementary Table S2: Biochemical profile of *Acintobacter* spp.

	Gram stain	catalase	oxidase	Nitrate	hemolysis	TSI	citrate	Arginine hydrolysis	VP	Urease	Galactose	Lactose	Mannitol	Sucrose	glucose
1	Gram -ve	+ve	-ve	-ve	-ve	k\k	+ve	+ve	-ve	-ve	+ve	-ve	-ve	-ve	+ve
2	Gram -ve	+ve	-ve	-ve	-ve	k\k	+ve	+ve	-ve	-ve	+ve	-ve	-ve	-ve	+ve
3	Gram -ve	+ve	-ve	-ve	-ve	k\k	+ve	w+ve	**V**	-ve	+ve	-ve	-ve	-ve	+ve
4	Gram -ve	+ve	-ve	-ve	-ve	k\k	W +ve	+ve	**V**	-ve	+ve	-ve	-ve	-ve	+ve
5	Gram -ve	+ve	-ve	-ve	-ve	k\k	+ve	+ve	-Ve	-ve	+ve	-ve	-ve	-ve	W +ve
6	Gram -ve	+ve	-ve	-ve	-ve	k\k	+ve	+ve	-ve	-ve	+ve	-ve	-ve	-ve	+ve
7	Gram -ve	+ve	-ve	-ve	-ve	k\k	+ve	+ve	-ve	-ve	**V**	+ve	-ve	-ve	+ve
8	Gram -ve	+ve	-ve	-ve	-ve	k\k	+ve	W +ve	-ve	**V**	+ve	-ve	-ve	-ve	+ve
9	Gram -ve	+ve	-ve	-ve	-ve	k\k	W +ve	+ve	-ve	**V**	+ve	-ve	-ve	-ve	W +ve
10	Gram -ve	+ve	-ve	-ve	-ve	k\k	W+ve	+ve	-ve	**V**	+ve	-ve	-ve	-ve	-ve
11	Gram -ve	+ve	-ve	-ve	-ve	k\k	W+ve	+ve	-ve	**V**	+ve	-ve	-ve	-ve	+ve
12	Gram -ve	+ve	-ve	-ve	-ve	k\k	+ve	+ve	-ve	-ve	**V**	-ve	-ve	-ve	-ve

13	Gram -ve	+ve	-ve	-ve	-ve	k\k	+ve	+ve	-ve	-ve	+ve	-ve	-ve	-ve	+ve

N.B. V:variable , W:weak, K/K: alkalien butt\alkalien slant,-VE :negative, +ve:postive

Supplementary Table S3: Results of biofilm formation by microtiter plate (OD600)

Isolates No.	Mean± ST	Biofilm degree
**Negative control** (Brain heart infusion broth only)	0.09231±0.004934258	Non-producing biofilm
A.baumannii (1)	0.359666667±0.039150521	Moderate
A.baumannii (2)	1.817233333± 0.037313045	Strong
A.baumannii (3)	0.1502± 0.01091192	Weak
A.baumannii (4)	0.093936667± 0.003447903	Non-producing biofilm
A.baumannii (5*)*	0.089866667±0.018681899	Non-producing biofilm
A.baumannii (6)	0.155733333±0.027713595	Weak
A.baumannii (7)	0.181463333± 0.003430894	Moderate
A.baumannii (8)	0.187333333± 0.003000556	Moderate
A.baumannii (9)	0.091823333± 0.001825824	Non-producing biofilm
**Positive control** P. aeruginosa ATCC 27853 reference strain	1.253066667± 0.061481894	Strong
